# P19. Immunomodulation of blasts in AML-patients (pts) with clinically approved response modifiers to improve anti leukaemic T-cell reactivity: an ex vivo simulation of the clinical situation

**DOI:** 10.1186/2051-1426-2-S2-P10

**Published:** 2014-03-12

**Authors:** H Schmetzer, Z Stankova, D Deen, A Hirn, Y Vokac, T Kroell, R Buhmann, A Hausmann, C Schmid, J Tischer

**Affiliations:** 1 University Hospital of Munich Grosshadern Med. Dept. 3, Department for Hematopoetic Cell Transplantations, Munich, Germany; 2 Helmholtz-Center Munich CCG HCT, CCG HCT, Munich, Germany; 3 Municipal Hospital Munich-Schwabing, Dept. for Hematopoetic Cell Transplantations, Munich, Germany; 4 Municipal Hospital Augsburg, Dept. for Hematopoetic Cell Transplantations, Munich, Germany

## 

Allogenic SCT/DLI are promising T-cell based therapies to cure AML-pts. Antileukaemic T-cell-reactivity has to be improved/re-established in pts in vivo. Ex vivo leukaemia-derived DC (DC_leu_) are the most effective antileukaemic T-cell-stimulators.

## Aim and methods

We generated DC_leu_ ex vivo from AML blasts from heparinised whole blood (‘**WB-DC’,** to simulate the in vivo situation) from 65 AML-pts in active stages of the disease using standard methods (‘Picibanil’, ‘MCM-Mimic’, ‘Ca-ionophore’, ‘IFNa’) or 11 minimalised cocktails (‘**WB-minicock-DC’,** combinations of 1-3 selected cytokines, antibiotics, bacterial lysates, or other clinically approved response-modifiers) and to correlate proportions of DC- or T cellsubsets and cytokine profiles with results with their ex vivo stimulatory capacity for antileukaemic T-cells and the pts' response to immunotherapy (SCT/DLI).

## Results

**1. Generation of DC:** we could identify 4 of 11 **mini-cocks**, that allowed the generation of DC/DC_leu_ from blast-containing WB-samples with at least one of the three methods. Some of the cocktails induced ex vivo blast-proliferation in individual pts. Proportions of DC-subtypes (e.g DC/DC_leu_/mature DC) were comparable to proportions generated with standard DC methods. **2. Antileukaemic functionality:** In 21 cases T-cells stimulated with 1 to 3 ‘**WB-minicock-DC**’ resulted in 56% cases with blast-lysis; in 6 pts 2-3 cocktails could be studied in parallel and in at least one of the cocktails a blastlysis could be achieved. Blast lysis (vs non-lysis) correlated with higher proportions of DC-subtypes: (DC,DC_leu_ blastconversion to DC_leu_), higher proportions of T-cell-subtypes (viable, CD8 Tcells), higher concentrations of IL-12 and IFNg but lower concentrations of IL-6 and IL-8 **3.Clinical correlation**: AML-pts successfully responding to immunotherapy (SCT or DLI therapy) presented with higher proportions of DC, DC_leu_ and CCR7^+^mature DC compared to pts without successful immunotherapy.

## Conclusion

DC/DC_leu_ can be generated regularly from MNC or WB and with at least 1 to 4 of 11 minicocks containing combinations of 1-3 selected, clinically approved response modifiers. T-cells stimulated with ‘WB-minicock-DC’ achieved antileukaemic function, although not with every cocktail. A patient-individual testing of the best cocktail as well as the achieved antileukaemic (ex vivo) function can contribute to define cocktails of responsemodifieres to be applicated to AML pts to achieve or sustain remission.

